# Sepsis and infection: Two words that should not be confused

**DOI:** 10.3389/fmed.2023.1156732

**Published:** 2023-03-09

**Authors:** Jean-Louis Vincent

**Affiliations:** Department of Intensive Care, Erasme Hospital, Université Libre de Bruxelles, Brussels, Belgium

**Keywords:** septic shock, organ dysfunction, personalized medicine, phenotypes, immune response

## Abstract

The underlying cause of sepsis is a dysregulated host response to infection, leading to multiple organ failure. Identifying sepsis is crucial because of the associated pathophysiological, practical, and therapeutic implications, which will determine where and how the patient should be managed. In the absence of an end-of-life decision to limit therapies, the patient should be admitted to the intensive care unit immediately. Importantly, not all patients with sepsis are the same and being able to better characterize them is important. The future will focus on phenotypes to characterize critically ill patients, with or without infection, to enable more appropriate targeting of therapeutic interventions.

## 1. Introduction

“Hello, I’d like you to take care of a patient who may be septic. This patient has a bad abdominal infection, and sepsis markers are elevated, I’m really worried about this infection…, I mean sepsis ….”

Over the years, there has often been confusion regarding the terms infection and sepsis. However, one word should not be replaced by another without careful thought as to the underlying meanings of both. Although we often use words interchangeably, and for many situations it is not important, in medicine, and particularly in the field of sepsis, choice of words can influence actions. Indeed, the word “sepsis” always indicates presence of an infection, but the word “infection” does not, on its own, indicate sepsis. One should keep in mind that the word “sepsis” originates from a word meaning “putrefaction” or “decay” in Greek: the word was used by the ancient Greeks to define a serious, usually fatal situation (1). Sepsis represents the most severe form of infection, so that intrinsically identification of sepsis means that some degree of organ dysfunction must be present: usually the patient is hypotensive, oliguric, and/or obtunded.

## 2. Organ dysfunction/Failure

Many different types of organ dysfunction can be present in patients with sepsis but six are (easily) quantifiable and have been included in most organ function scores, such as the sequential organ failure assessment (SOFA) (2). These are:

- Circulatory: quantified by presence of hypotension, need for vasopressor support, signs of the altered tissue perfusion, increased blood lactate levels.
- Respiratory: quantified by the alteration in gas exchange and often the need for respiratory support (invasive or non-invasive).
- Renal: quantified by an increase in blood creatinine and/or oliguria, sometimes need for renal replacement therapy.
- Hematologic: quantified by a low platelet count.
- Neurological: quantified by an altered mental status.
- Hepatic: quantified by increased bilirubin level without evidence of a regional biliary problem.

One could also consider endocrine dysfunction, perhaps using increased insulin requirements, and gastrointestinal dysfunction, characterized by difficulty with feeding, but these two organ functions are less easily quantified.

## 3. The pyramid of infection severity

**FIGURE 1:**
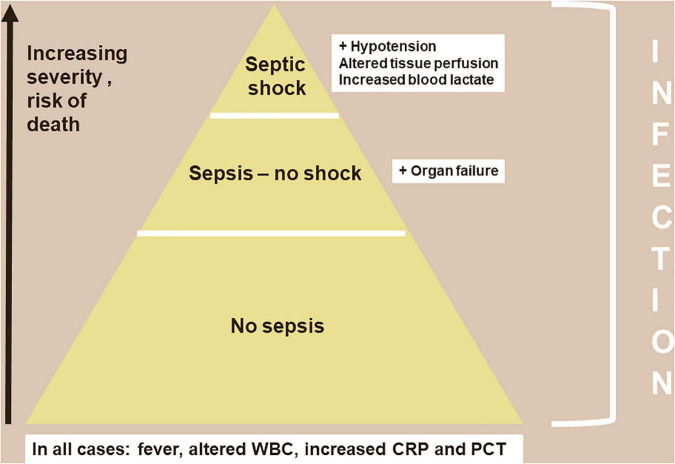
The pyramid of severity in infection.

The vast majority of infections are not associated with organ dysfunction and thus do not enter into consideration as sepsis; sepsis is only present when there is some organ failure attributed to the infection (Figure 1). Septic shock is said to occur when acute circulatory failure develops, typically characterized by a decrease in blood pressure associated with signs of altered tissue perfusion clinically manifest in three clinical “windows”: altered peripheral perfusion, with the skin typically mottled and cyanotic; altered brain perfusion, resulting in impaired mental status, characteristically with obtundation and disorientation; and altered renal perfusion with decreased urine output. A hallmark of shock is an increase in blood lactate levels, which are typically greater than 2 mEq/L (or mMol/L) (3). Lactate levels are now easily obtained by bedside analyzers.

The global incidence of sepsis is almost impossible to estimate given that many cases will occur outside of the hospital and many of those affected will not even seek primary healthcare consultation, notably in low income countries. A recent study, using data from the Global Burden of Diseases, Injuries, and Risk Factors Study 2017, estimated that some 49 million cases of sepsis occurred worldwide in 2017 and that sepsis accounted for almost 20% of global all-cause mortality (4). In high income countries, septic shock affects about 10% of patients admitted to the intensive care unit (ICU) (5), but estimates in other areas of the world are lacking. However, clearly, the global burden of sepsis and its impact on individuals, families, healthcare systems, and economies is enormous.

## 4. What does “sepsis” imply?

So, why does differentiating infection and sepsis matter? Identifying sepsis in a patient has important pathophysiological, practical, and therapeutic implications.

### 4.1. Pathophysiological

The pathophysiological mechanism underlying sepsis is best described as a dysregulated host response to infection (6, 7). As the pathophysiology of sepsis began to be explored and understood following the discovery of the link between microorganisms and infection at the end of the 19th century, the involvement of pro-inflammatory aspects of the immune system came to the fore (8), leading to attempts to suppress inflammation. However, later studies showed that some degree of acquired immunosuppression can develop early on during the disease process, so that although initially the response may be predominantly pro-inflammatory, it may rapidly “reverse” into a predominantly hyporeactive state, which may promote the development of secondary infections (7). Interestingly, the two types of response may occur concurrently in different parts of the body, and they may even coexist in neighboring cells (9). Moreover, the immune markers we measure in the blood may not reliably reflect what is actually happening in the organs. The sepsis response is thus highly complex, rendering our understanding difficult with current monitoring and available markers.

### 4.2. Practical

As shown earlier, identification of sepsis reflects a greater severity of the disease process. Immediate attention and treatment is required. Patients with sepsis should be admitted to the ICU for full resuscitative management and organ support, unless one of two situations is present:

1. The process is obviously under control and the patient is expected to improve rapidly.
2. A decision has been made to limit therapeutic interventions, thus preventing the implementation of organ support.

### 4.3. Therapeutic

**FIGURE 2:**
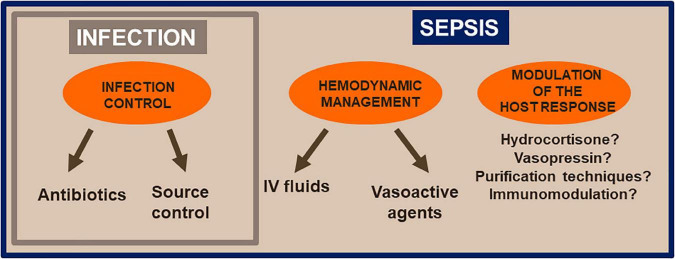
Factors of management of infection (small rectangle) and sepsis (large rectangle).

The treatment of an infection requires administration of appropriate antimicrobials (Figure 2). In the vast majority of cases, this will mean an antibiotic agent(s), but may involve anti-fungal or antiviral agents if indicated. Development and use of new molecular techniques, including multiplex polymerase chain reaction (PCR) assays, will help with early identification, and thus appropriate treatment, of infecting organisms. The source of infection may require drainage, which may be performed percutaneously, endoscopically or surgically. Organ support is generally not required in patients with infection, but if needed will be limited to some oxygen administration or intravenous fluid therapy. In sepsis, however, treatment moves beyond just infection control although this is still essential. The presence of organ failure necessitates more advanced organ support. This is particularly true in septic shock where patients may require large amounts of intravenous fluid in addition to vasopressor agents, such as noradrenaline. There may be a need for respiratory support, especially if there is associated acute respiratory distress syndrome (ARDS). There may also be a place for use of specific molecules that act on the host response. The first such molecule is hydrocortisone, which should be added to other treatments at a dose of 300 mg per day in patients with severe septic shock (10). Addition of vasopressin at a dose of 0.03 to 0.05 units/min can also be considered. The exact place of vasopressin is not yet well-defined, but it may be associated with reduced oedema formation and some renal protection (11). The use of blood purification techniques in sepsis, although supported by physiological rationale, remains investigational at present.

## 5. Mortality

Mortality rates in patients with infection are generally very low and are largely influenced by the type of infection and the degree of frailty of the patient, reflected primarily by age and comorbidities. Mortality rates associated with sepsis are usually around 30 to 35% (5). Mortality rates in patients with septic shock are higher at around 35–50%. Cited mortality rates are largely influenced by the type of study in which the data has been collected. Observational, epidemiological studies that include all patients with sepsis will include a number of patients in whom prognosis is associated with their degree of frailty, the presence of comorbidities, and/or end-of-life decisions; such studies are more reflective of the real-life situation. By contrast, interventional clinical trials evaluating the effects of a particular intervention are more restrictive in their entry criteria. Patients with therapeutic limitations (“do not resuscitate” orders) or with serious comorbidities are usually excluded from the trial, so that mortality rates may decrease below 40%. However, this process makes it difficult to demonstrate a decrease in mortality associated with the intervention and can thus result in a negative result when mortality is the primary end-point of the study, even though other morbidity endpoints may be improved.

## 6. How to characterize sepsis

Increasingly the need to characterize or stratify patients with sepsis is appreciated, so that treatments can be selected based on individual phenotypes reflecting immune status and likelihood of response (8, 12). An interesting approach, which combines the various aspects outlined herein, is the PIRO acronym (13). PIRO characterizes patients with sepsis according to four domains: predisposing factors including comorbidities; infectious aspects; the host response; and the type and degree of organ dysfunction/failure (Table 1). This approach has shown good performance for staging sepsis in different hospital wards and in different types of hospital (14).

**TABLE 1:** The predisposition, infection, response, and organ dysfunction (PIRO) system [adapted from (13)].

	Clinical	Laboratory/ Therapeutic
Predisposing factors	Age, genetic factors, Immunosuppression alcoholism, cirrhosis, …	Genotyping Cellular response (HLA-DR)…
Infection	Signs of pneumonia, meningitis, peritonitis, …	Chest Xray, CT scan… Microbiological data Bacterial DNA (PCR), …
Response	Fever, tachycardia, tachypnea, …	WBC count, CRP, PCT, specific sepsis markers…
Organ dysfunction	Circulatory failure, respiratory failure, renal failure, …	PaO_2_/FiO_2_ Urea, creatinine Platelet count, bilirubin…

CT, computed tomography; PCR, polymerase chain reaction; CRP, C-reactive protein; PCT, procalcitonin; WBC, white blood cell.

## 7. Phenotypes

Rather than targeting sepsis *per se* with our novel therapies, a new strategy is to focus on phenotypes, with or without infection. This will enable us to increase homogeneity, and concentrate on the right pathophysiologic pathway, whilst keeping enough patients enrolled in clinical trials. Interventions will target specific abnormalities, e.g., thrombomodulin in coagulopathy, anti-inflammatory strategies in patients with elevated CRP and interleukin (IL)-6 levels, adrenomedullin antibody administration in patients with high adrenomedullin levels, and so on. There is a need for precision immunotherapies guided by appropriate biomarkers.

## 8. Conclusion

Although the terms infection and sepsis are sometimes used interchangeably, they do not refer to the same condition. Sepsis is the most severe form of infection, when the host response becomes dysregulated, so that organ dysfunction develops. Identification of sepsis in a patient has important pathophysiological, practical, and therapeutic implications, which will determine where and how the patient should be managed most appropriately. Increasingly, the complex individual nature of sepsis in terms of immune response and response to therapy is being recognized, and the ability to better characterize patients according to specific phenotypes will enable more targeted selection for clinical trials and ultimately for personalized therapies.

## Data availability statement

The original contributions presented in this study are included in the article/supplementary material, further inquiries can be directed to the corresponding author.

## Author contributions

The author confirms being the sole contributor of this work and has approved it for publication.
